# Prediction of risk and incidence of dry eye in critical patients^1^

**DOI:** 10.1590/1518-8345.0897.2689

**Published:** 2016-05-17

**Authors:** Diego Dias de Araújo, Natália Gherardi Almeida, Priscila Marinho Aleixo Silva, Nayara Souza Ribeiro, Andreza Werli-Alvarenga, Tânia Couto Machado Chianca

**Affiliations:** 2 Doctoral Student, Escola de Enfermagem, Universidade Federal de Minas Gerais, Belo Horizonte, MG, Brazil. Professor, Universidade Estadual de Montes Claros, Montes Claros, MG, Brazil; 3 RN, Secretaria Municipal de Saúde, Prefeitura Municipal de Belo Horizonte, Belo Horizonte, Brazil; 4 Undergraduate Student in Nursing, Escola de Enfermagem, Universidade Federal de Minas Gerais, Belo Horizonte, MG, Brazil; 5 PhD, Adjunct Professor, Departamento de Enfermagem Básica, Escola de Enfermagem, Universidade Federal de Minas Gerais, Belo Horizonte, MG, Brazil; 6 PhD, Full Professor, Departamento de Enfermagem Básica, Escola de Enfermagem, Universidade Federal de Minas Gerais, Belo Horizonte, MG, Brazil

**Keywords:** Dry Eye Syndromes, Corneal Diseases, Intensive Care Units, Nursing, Nursing Diagnosis

## Abstract

**Objectives:**

to estimate the incidence of dry eye, to identify risk factors and to establish a risk prediction model for its development in adult patients admitted to the intensive care unit of a public hospital.

**Method:**

concurrent cohort, conducted between March and June, 2014, with 230 patients admitted to an intensive care unit. Data were analyzed by bivariate descriptive statistics, with multivariate survival analysis and Cox regression.

**Results:**

53% out of 230 patients have developed dry eye, with onset mean time of 3.5 days. Independent variables that significantly and concurrently impacted the time for dry eye to occur were: O2 in room air, blinking more than five times per minute (lower risk factors) and presence of vascular disease (higher risk factor).

**Conclusion:**

dry eye is a common finding in patients admitted to adults intensive care units, and care for its prevention should be established.

## Introduction

Patients in very critical conditions are normally admitted to Intensive Care Units (ICUs). Most of the time these patients are sedated, in a coma, with Mechanical Ventilation (MV), taking several medications and with compromised ocular protection mechanisms^(^ 1 ^-^ 8 ^)^.

In ICUs, so far, little importance has been attributed to the care of damage or injury related to the visual perception of critical patients, occurring for several causes, since its approach requires knowledge and participation of a multidisciplinary team, and care for reducing ocular problems^(^ 4 ^,^ 8 ^-^ 9 ^)^. In addition, ICUs favor assistance to systems considered vital (cardiovascular, respiratory and neurological).

The dysfunction of the tear film, known as dry eye, is a multifactorial alteration of tears and the ocular surface that results in symptoms of discomfort, visual disturbances and instability of the tear film, with potential damage to the ocular surface. The problem is followed by an increase in the osmolarity of the tear film, and ocular surface inflammation^(^ 10 ^)^.

The nursing diagnosis of risk of dry eye is defined as "risk of ocular discomfort and damage to the cornea and conjunctiva due to the reduced amount or quality of tears to moisten the eye". The risk factors for the problem in patients admitted to ICUs involve sedation, environmental factors (air conditioning and low humidity) related to the treatment (side effects of pharmaceutical agents such as diuretics, analgesics, sedatives and neuromuscular blocking agents), mechanical ventilation therapy, neurological lesions with sensory or motor loss, and damage to the ocular surface^(^ 9 ^)^.

The preventive approach to ocular care is of utmost importance for patients admitted to ICUs. The absence of specific care for prevention of dry eye can negatively impact the lives of patients, both during hospitalization and after discharge from the ICU, for generating discomfort and ocular damage that may limit daily activities and compromise the quality of life.

Only a few studies support dry eye in patients admitted to ICUs. We were able to find one study^(^ 4 ^)^ related to the problem in critical patients, however, it was about dry eye prevention, lacking the identification of incidence and risk factors of the problem.

This research is justified by the need for knowledge about the dry eye problem, determination of its incidence and risk factors in critical patients, and thus implements practices based on scientific evidence, for prevention and treatment of this involvement identified in patients admitted to ICUs. It should be noted that no studies that specifically cover the problem were identified, although NANDA I^(^ 9 ^)^ has approved the Nursing Diagnosis of risk of dry eye in the 2012-2014 edition.

This study aims to estimate the incidence of dry eye, to identify risk factors and to establish a risk prediction model for its development in adult patients admitted to the intensive care unit of a public hospital.

## Methods

This is a concurrent cohort study, conducted in an ICU for adult patients of a public teaching hospital in Belo Horizonte, Minas Gerais. Currently, in this hospital, 30 intensive care beds intended for adults are available to the community.

Sample size calculation was carried out using the infinite population formula, by conservative criterion, since the studied population was unknown. In the estimation of the sample we considered the infinite population, confidence degree of 95%, margin of error of 6.5%, and proportion of interest of 55.1% in the incidence of lesions in the punctate cornea^(^ 8 ^)^, resulting in a minimum sample calculation of 225 patients.

The inclusion criteria were: to have 18 years or more, to not present dry eye at the time of admission, to remain hospitalized in intensive care for at least 24 hours, to consent to participate in the research or have their participation authorized by the responsible person through a free and informed consent form.

The target population of this research consisted of 258 patients hospitalized in the ICU between March and June, 2014. Eight out of 258 patients were excluded because their relatives did not allow the participation in the study, one for being underage, and the other 19 for having dry eye diagnosis at the time of admission to the unit. Therefore, after the application of the inclusion and exclusion criteria, a total of 230 patients was sampled.

For data collection we used an evaluation tool for admission, which included sociodemographic and clinical information, and risk factors for the development of dry eye. Twenty-four hours after the admission, the patients were evaluated with the instrument of daily developments, which included clinical data and risk factors for the development of dry eye, identified in the literature^(^ 1 ^-^ 23 ^)^.

The dependent variable was the time for the occurrence of dry eye in patients admitted to adults ICU. The independent variables, selected in literature^(^ 1 ^-^ 23 ^)^, were: age, gender, origin unit, Sepsis Related Organ Failure Assessment (SOFA), Acute Physiology and Chronic Health Evaluation (APACHE II), Therapeutic Intervention Scoring System (TISS 28), type of patient, death, postoperative time, days of hospitalization, referral to another ward or hospital, medical diagnosis, sedation, score in Ramsay sedation scale, score in the Glasgow Coma scale (GCS), tracheal intubation, tracheostomy (TQT), Mechanical Ventilation (MV), days with MV, MV type, Fraction of Inspired Oxygen (FiO2), End-expiratory Pressure (PEEP), orotracheal tube fixing, Noninvasive Ventilation (NIV), NIV time, Oxygen (O_2_) in room air, O_2_ by Nasal Catheter (NC), use of macronebulization, oxygen flow, blinking rate per minute, eyeball exposure, oedema, conjunctival hemorrhage, severity of corneal injury, medicines, oral diet allowed, feeding tube, nutritional status, accumulated fluid balance (FB), positioning (degree of elevation of the headboard), and white blood cells.

Before data collection, the nurse researcher was trained to evaluate the cornea by a nurse with experience and training in corneal evaluation of the critically ill patients. The training consisted in theoretical explanation about corneal injury and practical training of eye evaluation, in addition to reading articles and texts on the subject. This nurse was considered the gold standard for performing corneal evaluation due to the experience with assistance, research and publication in the area. We found Kappa coefficient of 0.84 between the nurse researcher and the nurse considered expert, i.e., an almost perfect agreement.

Data collection was carried out every day of the week by the nurse researcher until the patient developed an outcome, were discharged from ICU, transferred or passed away. To evaluate the tear volume we used the Schirmer I test, which consists in the installation of a strip of Whatman no. 41 or 50, with 5 mm in width and 35 mm in length, with folded extremity (about 5 mm), attached to the bottom of the lower eyelid bag in the temporal part (the outer corner of the lower eyelid). After 5 minutes, the tape was removed, measured, and the extension of the moistened part was noted. For corneal evaluation we installed a drop of fluorescein in each eye of the patient, and after 1 to 2 minutes, under low light conditions, the cornea was examined with the aid of an ophthalmoscope with cobalt blue light filter and magnifying glass, for best viewing of possible corneal changes. Data were immediately noted in the data collection instrument.

In the treatment of the data, we performed double typing in Epi Info program, version 3.5.1, and after verifying the consistency of the data they were exported to the Statistical Package for Social Science (SPSS), version 19.0.

In the analysis, we used simple frequencies, measures of central tendency (mean and median), and measures of variability (standard deviation). The incidence (global incidence and incidence rate) of dry eye and risk factors were determined. For analysis of the potential risk factors with the time to the occurrence of dry eye in patients hospitalized in ICU, we used bivariate analysis for the variables studied, from the survival analysis. With that, we obtained the relation between each independent variable and the outcome variable (time before the occurrence of dry eye), being measured the strength of the association by the Hazard Ratio (HR), considering the confidence interval (CI) of 95%. To identify surveyed covariates that exerted influence on the time from the monitoring to the outcome, we used the Cox regression model. Variables whose p value was ≤ 0.25 in the bivariate analysis were included in the multivariate analysis model. We performed the global adjustment of the model by the probability ratio test, estimated the survival function, failure rate regarding the time before the occurrence of dry eye, and risk proportionality test.

The study is in accordance with Resolution 466/12, which provides for research with human beings. The project was referred to the Ethics and Research Committee of the Federal University of Minas Gerais and obtained a favorable opinion under the CAAE Protocol - 15616313.4.0000.5149.

## Results

Among the 230 patients, 122 presented dry eye. The global incidence of dry eye was, therefore, of 53% in the period of the study. The incidence rate of dry eye was of 0.184 cases/patient a day (5.51 cases/patient per month), ranging from 0.153 cases/patient a day (4.58 cases/patient per month) to 0.219 cases/patient a day (6.58 cases/patient per month), with 95% confidence.

Most (55.7%) were male, mean age of 59 years (SD ± 19.2), median of 62 years, with minimal variability of 18 years and a maximum of 97 years. Of the total patients (230), 36% were sedated. Intubation was used in 110 (48%); tracheostomy in 6 (2.6%), and mechanical ventilation in 114 (50%). Among the patients studied, 8% passed away. The seriousness of the medical condition of the patients was evaluated by the instruments, SOFA, APACHE II and TISS 28, applied in the first 24 hours of the patient's hospitalization in ICU. On average, they had a 4.9 SOFA, 20 APACHE II and 31 of 28 TISS. For admission to the ICU, vascular diseases were the most frequent (27%).

More than half of patients blinked the eyes more than five times per minute (51.3%), and 49.2% had the eyeball exposed (lagophthalmos).

Among the patients, 53% showed positive Schirmer I test, and 54.3% presence of corneal injury. Of these, 52% showed punctate-type injury and 6% corneal ulcer; 50% did not show injuries in both eyes. However, 30% showed punctiform epithelial erosions, involving the lower third of the cornea of the left and right eyes.

On average, for admission, the patients presented a moistened extension of the Whatman Strip of 14.6 mm and 12.9 mm in the left eye, in the Schirmer test I, during the study. For the right eye, for admission, the result was of 15 mm of moistened extension, and 13.1 mm during the study.

In the bivariate analysis, we obtained variables that showed statistical significance (p≤0.25) over time until the occurrence of dry eye. For multivariate analysis, 40 variables were eligible, of which 30 showed statistical significance (p<0.05), presented in Table 1.

**Table 1:** - Variables with association with the time until occurrence of dry eye. Belo Horizonte, MG, Brazil, 2014

Variables	Group	Dry eye	Hazard Ratio (HR) (CI 95%)	p value					
		Yes		No					
		n	%		n	%			
Sedation	No	51	41.8		96	88,9	2.10 (1.46 - 3.02)	< 0.001	
	Yes	71	58.2		12	11.1			
Intubation	No	27	22.1		93	86.1	3.16 (2.06 - 4.85)	< 0.001	
	Yes	95	77.9		15	13.9			
Mechanical Ventilation	No	24	19.7		92	85.2	3.40 (2.17 - 5.32)	< 0.001	
	Yes	98	80.3		16	14.8			
O2 on room air	No	116	95.1		50	46.3	0.12 (0.05 - 0.29)	< 0.001	
	Yes	6	4.9		58	53.7			
O2 by nasal catheter	No	103	84.4		46	42.6	0.27 (0.16 - 0.44)	< 0.001	
	Yes	19	15.6		62	57.4			
Oral diet allowed	No	62	50.8		37	34.3	0.43 (0.30 - 0.63)	< 0.001	
	Yes	60	49.2		71	65.7			
Feeding tube	Enteral feeding	45	75.0		11	15.5	3.01 (1.64 - 5.49)	< 0.001	
Blinking	Up to five times per minute	101	82.8		11	10.2	0.16 (0.10 - 0.26)	< 0.001	
	More than five times per minute	21	17.2		97	89.8			
Eyeball exposure	No	62	50.8		104	96.3	2.43 (1.70 - 3.48)	< 0.001	
	Yes	60	49.2		4	3.7			
Oedema	No	12	9.8		40	37.0	2.39 (1.31 - 4.34)	0.004	
	Yes	110	90.2		68	63.0			
Oedema site	Chemosis - left eye	45	36.9		9	8.3	1.79 (1.24 - 2.59)	0.002	
	Chemosis - right eye	45	36.9		9	8.3	1.79 (1.24 - 2.59)	0.002	
	MMII	70	57.4		34	31.5	1.47 (1.03 - 2.11)	0.033	
	Anasarca	9	7.4		1	0.9	3.05 (1.53 - 6.09)	0.001	
Anticoagulants	Yes	82	67.2		84	77.8	0.64 (0.43 - 0.93)	0.022	
Hypnotics/sedatives/anxiolytics	Yes	71	58.2		20	18.5	1.58 (1.10 - 2.27)	0.013	
Antihypertensives	Yes	19	15.6		32	29.6	0.53 (0.32 - 0.87)	0.013	
Analgesic	Yes	22	18.0		43	39.8	0.62 (0.39 - 0.99)	0.048	
SOFA*†	Yes	6.8	3.7		4.0	3.5	1.10 (1.05 - 1.15)	< 0.001	
APACHE II*‡	Yes	25.4	7.5		19.0	9.7	1.03 (1.01 - 1.04)	< 0.001	
TISS 28*§	Yes	35.8	8.1		26.7	8.7	1.06 (1.03 - 1.08)	< 0.001	
Postoperative time*	Yes	2.9	1.0		2.6	1.1	0.44 (0.31 - 0.63)	< 0.001	
Hospitalization Time*	Yes	3.0	0.9		2.8	1.2	0.05 (0.02 - 0.13)	< 0.001	
Glasgow Coma Scale*	Yes	8.9	4.2		14.2	1.3	0.84 (0.80 - 0.89)	< 0.001	
Mechanical Ventilation Time*	Yes	3.2	1.2		2.1	1.0	0.64 (0.50 - 0.82)	< 0.001	
O2 flow catheter*	Yes	2.3	1.4		1.6	0.7	1.41 (1.01 - 1.97)	0.039	
Anticoagulants*	Yes	82	67.2		84	77.8	0.64 (0.43 - 0.93)	0.022	
Hypnotics/sedatives/anxiolytics*	Yes	71	58.2		20	18.5	1.58 (1.10 - 2.27)	0.013	
Antihypertensives*	Yes	19	15.6		32	29.6	0.53 (0.32 - 0.87)	0.013	
Fluid balance/Daily developments (Positive)*	Yes	894.3	978.1		595.2	1022.0	1.00 (1.000003 - 1.0004)	0.046	

*Variables in which n corresponds to the mean and % to the standard deviation †SOFA - Sepsis-related Organ Failure Assessment ‡APACHE II - Acute Physiology and Chronic Health disease Classification System II §TISS 28 - Therapeutic Intervention Scoring System

Risk prediction model (multivariate analysis) among the demographic and clinical factors identified, O_2_ in room air, blinking more than five times per minute, and presence of vascular disease impacted significantly and concurrently until the occurrence of dry eye.

Patients who remained in O_2_ for room air presented probability of occurrence of dry eye regarding the risk group which was not in O_2_ by room air, at any time, 66% lower (HR=0.34), ranging from 13% to 87%, with 95% confidence (p=0.025).

Patients who blinked their eyes more than five times per minute showed probability of occurrence of dry eye, at any time, 75% lower (HR=0.25) in relation to those who blinked their eyes up to five times per minute, ranging from 57% to 86%, with 95% confidence (p<0.001).

For patients who have medical diagnosis of vascular disease when admitted to the ICU, the risk of dry eye, at any time, was 1.56 (HR=1.56) higher compared to those who did not have such medical diagnosis. The risk ranged from 1.03 to 2.38 times, with 95% confidence (p=0.037).

Among the studied patients, we observed that dry eye occurred only from the second day, considering that between the third and the fourth days of hospitalization 50% of patients presented an outcome, according to the estimated model. The mean time determined for the occurrence of dry eye appearance was 3.5 days.

The risk of failure, determined to the development of dry eye, was of 0.1 time until the second day, achieving 0.5 times on the third day, 1.1 time on the fourth day, and 2.6 times in the sixth day of hospitalization.

In Table 2 we observed that the values of the Pearson correlation coefficient (p) are all close to zero. In addition, we also observed that both the global test and the tests for each variable do not show evidence for rejecting the null hypothesis of proportional risks.

**Table 2:** - Test of proportionality of risks. Belo Horizonte, MG, Brazil, 2014

Variables	p*	T^†^	p value
O_2_ per room air	-0.20126	3.69	0.0547
Blinking more than five times per minute	0.12170	1.89	0.1691
Vascular disease	0.02095	0.05	0.8185
GLOBAL	NA	5.52	0.1373

*Pearson correlation coefficient estimated between the Schoenfeld standardized residues and the variable time response before the occurrence of the outcome †Statistics of the test with Chi-square distribution, 3 degrees of freedom, approximately

## Discussion

In previous studies^(^ 11 ^-^ 15 ^)^, conducted with patients in outpatient units, dry eye prevalence ranged from 10.8% to 57.1%. However, these studies were conducted with a profile of patients different from the one of the patients who participated in this study. The only study^(^ 4 ^)^ on dry eye in critical patients found in the literature is a randomized clinical trial, comparing interventions for the prevention of the problem. We highlight that such study was conducted with a small sample (18 patients), and in a reality different from Brazil's.

As the reduction in the amount or the quality of tears can be the beginning of major alterations in the ocular surface, the prevention of dry eye becomes essential for critically ill patients. This problem can be identified and prevented by the nurses, who have nursing interventions to assist in the reduction of ocular complications.

Studies^(^ 16 ^-^ 17 ^)^ suggest that alterations in the ocular surface are highly prevalent, especially in the early days of hospitalization. It is estimated that the mean for the development of corneal injury is between 24 hours and 8.9 days^(^ 1 ^,^ 6 ^-^ 8 ^,^ 18 ^)^. In the study^(^ 4 ^)^ that specifically addressed dry eye, the mean for the emergence of the problem was approximately three days, but this time was estimated after the intervention implementation.

The emergence of dry eye is estimated at a relatively small time interval. Thus, once the patient is admitted to the ICU, ocular conditions should be evaluated, and nursing interventions implemented to prevent possible ocular complications that may impact negatively on the lives of these patients during the hospitalization or after discharge of the unit.

Among the variables that showed a significant association (p<0.05) with time until the occurrence of dry eye and that predispose higher risk for developing dry eye (HR≥ 1), authors^(^ 1 ^-^ 10 ^)^ point out as possible risk factors: values obtained with APACHE II, TISS 28, hospitalization time, intubation, Mechanical Ventilation (MV), score in the Glasgow Coma scale, MV time, hypnotics/sedatives/anxiolytics, fluid balance/daily evolution (positive), lagophthalmos, chemosis, and anasarca.

Regarding the variables identified as factors that predispose lower risk of developing dry eye in critical patients, by presenting HR<1, such variables may be related to the better clinical pattern of patients, since they would already be taking the allowed diet, without intubation, breathing with the aid of mechanical ventilation, with adequate blinking reflex, and may complain of pain. However, we highlight that medications like anticoagulants, analgesics, and antihypertensive drugs are commonly administered in critical patients, hospitalized in ICUs and, although a statistically significant association was found, further studies are needed to evaluate whether there is a direct causal relation between the use of such medicines and the development of dry eye.

We also highlight that, in this study, lagophthalmos (eyeball exposure) was identified as the main risk factor for changes in the ocular surface. The datum is corroborated by other studies^(^ 17 ^,^ 21 ^-^ 22 ^)^, in which the patients had lagophthalmos frequency ranging from 31% to 54%. The fact is also pointed out by a study^(^ 23 ^)^ in which this risk factor showed statistical significance (p=0.001), corroborating the results of this research. Although the study^(^ 4 ^)^ did not find statistically significant results which specifically addressed the dry eye, the authors emphasize that lagophthalmos is one of the most important predictive factors for the occurrence of dry eye in critical patients.

Sedative drugs and mechanical ventilation were also identified as important risk factors for dry eye, and showed statistical significance (p=0.001). These data are confirmed by other studies^(^ 19 ^,^ 22 ^-^ 23 ^)^. Both risk factors are important for the problem, despite the results of the study^(^ 4 ^)^, conducted specifically on dry eye, showing no statistical significance.

Three covariates showed statistical significance (p<0.05) for the time until the occurrence of dry eye. Thus, patients who blinked their eyes more than five times per minute (HR=0.25), and in room air (HR=0.34), presented lower risk of dry eye. We can infer that the patients of the group with lower risk had a clinical profile less severe than patients that presented outcome.

Patients hospitalized with diagnosis of vascular disease (HR=1.56) showed higher risk of dry eye, which may be explained by the fact that these patients are usually sedated, in a coma, breathing with the aid of mechanical ventilation, using various medicines and with compromised ocular protection mechanisms. Thus, in a way, these patients were exposed to various factors associated with ocular surface changes, and the emergence of dry eye.

Regarding the function of failure, we found close temporal relation between dry eye and hospitalization in the ICU, i.e. the longer the hospitalization time, the greater the risk of developing outcome in critically ill patients.

The risk factors identified in the study are related to the medical condition and treatment of the patients. Although the nursing staff cannot change these factors, strategies for early identification of risk of dry eye can be adopted, as well as preventive measures to avoid further ocular injury that can be implemented from the ocular evaluation, using accurate tests such Schirmer I, fluorescein, and identification of risk factors related to the problem.

Some risk factors described in NANDA I^(^ 9 ^)^ have been validated in this study as: treatment side effects, neurological lesions with sensory loss or reflex motor (lagophthalmos, absence of spontaneous blinking reflex due to reduced consciousness or other medical conditions), and mechanical ventilation therapy. Certain factors presented to this diagnosis, and constants in the taxonomy were not validated for not presenting statistically proven relevance, for instance, the case of female patients with autoimmune diseases, aging, and hormone use.

However, other factors in the diagnosis could not be validated as well since they involve missing aspects in patients of this sample such as lifestyle, history of allergies, use of contact lenses, environmental factors, and the place where they live. We suggest multicentric research decrease in different populations, for comparing different realities.

The model obtained was considered valid to describe the relation between the time before the occurrence of dry eye and associated risk factors, in addition to anticipate which critically ill patients show risk of dry eye. Furthermore, the analysis of proportional risks tests shows that the model is appropriate, considering that the proportional risks assumption was met, thus determining the implementation of nursing care for its prevention.

A limitation of this study was its performance with a particular profile of patients, showing the need for multicentric studies in different populations to establish its external validity.

## Conclusion

From the results, we can verify that dry eye in patients admitted to adults ICUs is a common finding, exposed to a set of internal and external risk factors that can collaborate for the emergence of the problem.

After bivariate analysis and multivariate analysis adjustment step, among the demographic and clinical factors identified, those that remained as better predictors for the phenomenon studied were: O_2_ in room air, blinking more than five times per minute and presence of vascular disease.

The early recognition of risk factors for dry eye and, consequently, the adoption of preventive measures will certainly reduce the probability of ocular surface changes in critically ill patients.

It is recommended that the investigation of the risk factors described in NANDA I and which could not be validated according to the profile of the sample studied, such as: lifestyle, history of allergies, contact lenses, environmental factors and place where the patient lives. In addition, we need studies that allow establishing what is the best nursing care for the prevention of the problem, particularly regarding critically ill patients.

We believe that this study may contribute to reflect on the relevance of the dry eye problem in critical and non-critical patients, in addition to a greater awareness and appreciation of the importance of eye care in patients admitted to adults ICUs, being a fundamental aspect for higher quality nursing care.
